# Effect of Caffeic Acid Phenethyl Ester on Vascular Damage Caused by Consumption of High Fructose Corn Syrup in Rats

**DOI:** 10.1155/2016/3419479

**Published:** 2016-03-02

**Authors:** Aburrahman Gun, Mehmet Kaya Ozer, Sedat Bilgic, Nevin Kocaman, Gonca Ozan

**Affiliations:** ^1^Department of Pharmacology, Faculty of Medicine, Firat University, Elazığ, Turkey; ^2^Department of Pharmacology, Faculty of Medicine, Adıyaman University, Adıyaman, Turkey; ^3^Department of Biochemistry, Vocational School of Health Services, Adıyaman University, Adıyaman, Turkey; ^4^Department of Histology, Faculty of Medicine, Firat University, Elazığ, Turkey; ^5^Department of Biochemistry, Faculty of Veterinary, Firat University, Elazığ, Turkey

## Abstract

Fructose corn syrup is cheap sweetener and prolongs the shelf life of products, but fructose intake causes hyperinsulinemia, hypertriglyceridemia, and hypertension. All of them are referred to as metabolic syndrome and they are risk factors for cardiovascular diseases. Hence, the harmful effects of increased fructose intake on health and their prevention should take greater consideration. Caffeic Acid Phenethyl Ester (CAPE) has beneficial effects on metabolic syndrome and vascular function which is important in the prevention of cardiovascular disease. However, there are no known studies about the effect of CAPE on fructose-induced vascular dysfunction. In this study, we examined the effect of CAPE on vascular dysfunction due to high fructose corn syrup (HFCS). HFCS (6 weeks, 30% fed with drinking water) caused vascular dysfunction, but treatment with CAPE (50 micromol/kg i.p. for the last two weeks) effectively restored this problem. Additionally, hypertension in HFCS-fed rats was also decreased in CAPE supplemented rats. CAPE supplements lowered HFCS consumption-induced raise in blood glucose, homocysteine, and cholesterol levels. The aorta tissue endothelial nitric oxide synthase (eNOS) production was decreased in rats given HFCS and in contrast CAPE supplementation efficiently increased its production. The presented results showed that HFCS-induced cardiovascular abnormalities could be prevented by CAPE treatment.

## 1. Introduction

Fructose consumption in the form of high fructose corn syrup or sucrose, particularly as sweeteners of carbonated beverages, has increased dramatically in the last 30 years [1–3]. The consumption of food and beverages made of corn originated fructose plays an important role in the chronic diseases of childhood and adolescence in the modern world [2, 4–8]. As a result of the epidemiological and the experimental studies, it was suggested that the high fructose nutrients, especially along with physical immobility and consumption excess, could play a significant role in the development of chronic diseases (such as hypertension, obesity, metabolic syndrome, kidney disease, and calculi). And it was particularly pointed out that the problem underlying this situation might be the fructose [2, 9]. Studies have been conducted on the relationship between fructose consumption and obesity and the metabolic syndrome and hypertension [2, 10–14].

Caffeic Acid Phenethyl Ester (CAPE) is an active component of propolis substance which regulates the immune system and the immunostimulatory effect of which is known and whose structure is similar to the flavonoids produced by the honey bee [15]. It is known that CAPE has hepatotoxicity protective, anti-inflammatory, antioxidant, antiviral, immunomodulatory, neuroprotective, and cytostatic effects [16, 17]. Its antiinflammatory effect is more apparent compared to other components of propolis, for it strongly modulates the arachidonic acid cascade [15]. CAPE has got two annular structures [18]. One of these annular structures is carrying functional two OH-groups showing almost all chemical properties of CAPE molecules. These hydroxyl groups take and give actively the electrons and thus show oxidant and reductant characteristics. It has got lipophilic properties due to carrying very long carbon groups of aromatic and aliphatic structure [19, 20]. It is a potential inhibitor of enzymes such as ornithine carboxylase, 5-*α* reductase, protease, cyclooxygenase, lipoxygenase, and xanthine oxidase HIV-1 integrase [19, 21–23]. It specifically and strongly inhibits the activation of the Nuclear Factor Kappa-B (NF-*κ*B), which is a nuclear transcription factor [24]. That is why the CAPE can be used therapeutically.

Our aim of this study is to investigate whether CAPE (50 micromol/kg, i.p. for the last two weeks) has any therapeutic effect with respect to vessel damage that occurs due to consumption of fructose with drinking water (30% for six weeks with drinking water), or not. We did not find such a study in the literature research. To demonstrate this, we planned to evaluate the vessel in two aspects: the first part of the plan was to measure the capacity of endothelial nitric oxide synthase (eNOS) by using the immunohistochemistry method to understand the failure in the responses of the possible vasospasm and relaxation; the second part of the plan was to see and evaluate the responses of phenylephrine and acetylcholine in the thoracic aorta in vitro given high fructose corn syrup (HFCS) and HFCS + CAPE.

## 2. Material and Methods

### 2.1. Chemicals

Chemicals were purchased from Sigma Chemical Co. (St. Louis, MO) and Merck (Rahway, NJ) unless otherwise stated. HFCS (F42) was obtained from Sunar Grup (Turkey). HFCS contains approximately 42% fructose, 53% glucose, and 5% higher saccharides in the syrup of total solids. CAPE was obtained from Herb-Tech (ROC). eNOS was obtained from Thermo Scientific.

### 2.2. Animals and Diets

All animal procedures were approved by the Ethical Animal Research Committee of Firat University (04.04.2013/51). Eighteen Sprague Dawley rats aged 8 weeks were housed in temperature-controlled rooms (20–22°C) under a 12 h light-dark cycle. The rats were fed with standard commercial chow diet ad libitum. The rat diet was composed of 62% starch, 23% protein, 4% fat, 7% cellulose, standard vitamins, and salt mixture. After acclimation for 1 week, the rats were randomly divided into three groups (six rats in each group): control; HFCS (30%, 6 weeks with drinking water), and HFCS (30%, 6 weeks with drinking water) + CAPE (50 micromol/kg, i.p. for 2 weeks). The concentration of CAPE was assigned from our previous in vivo observations [25]. HFCS (42% fructose and 53% glucose) was prepared as 30% (w/v) solutions and administered to rats in drinking water for 6 weeks either in the presence or absence of CAPE. These concentrations of HFCS were determined according to the sugar content of numerous soft drinks in which sugar ranges from 7% to 15% [26]. Animals consumed fluids ad libitum. Food and water intakes as well as body weights were recorded weekly during the follow-up. After 6 weeks, the rats were euthanized by CO_2_.

### 2.3. Measurement of Systolic Blood Pressure

Systolic blood pressure (SBP) was measured by tail-cuff method (tail-cuff, BIOPAC Systems, NIBP200A) in the conscious, prewarmed, and restrained rats. The blood pressure readings were repeated four times between 9:00 a.m. and noon (12:00 a.m.).

### 2.4. Measurement of Lipids, Glucose, Homocysteine, and Uric Acid

Cardiac blood samples of nonfasted rats were centrifuged at 4°C and 10.000 g for 30 min. Serum samples were immediately stored at −85°C until the samples were assayed. High density lipoprotein (HDL), very low density lipoprotein (VLDL), total cholesterol, triglyceride, homocysteine, uric acid, and glucose levels were determined by standard enzymatic techniques.

### 2.5. Preparation of Thoracic Aortas and Measurement of Vascular Reactivity

The thoracic aortas of the rats were isolated and immediately placed into cold Krebs solution of the following composition (mmol/L): NaCl 118, KCl 4.73, KH_2_PO_4_ 1.2, MgSO_4_7H_2_O 1.2, CaCl_2_ 2.5, NaHCO_3_ 25, and glucose 10.1. The aortic rings of 3-4 mm in length were mounted in a 10 mL organ bath containing Krebs solution at 37°C and aerated with 95% O_2_ and 5% CO_2_. Four to six rings were prepared from each aorta and studied in parallel. A caution was exercised to preserve the endothelial layer during preparation of the aortic rings. The isometric forces of the rings were measured by using force displacement transducers (EMKA, Paris). In the aortic rings of rats, a passive stretch of 1 g which was determined to be optimal tension for maximal responsiveness to phenylephrine (10^−6^ M) that produced a tension of less than 1 g was not included in the experiments. The preparations were allowed to equilibrate for approximately 1 h with an exchange of bathing solution every 15 min. The presence of the endothelium was tested functionally by applying acetylcholine (10^−6^ M) on phenylephrine (10^−6^ M) precontracted aortic rings and preparations demonstrating <70% relaxations in the control group were discarded [27]. The cumulative concentration-response curves of phenylephrine (10^−9^ M–10^−4^ M) were constructed in aortic rings. The relaxing effects of acetylcholine (10^−9^ M–10^−4^ M) were studied in arterial rings constricted submaximal with phenylephrine.

### 2.6. Preparation of Thoracic Aortas and Measurement of Vascular e-NOS Reactivity

The avidin-biotin-peroxidase complex (ABC) was used for immunohistochemistry. Vessel tissues taken from the fixing solution were subjected to routine paraffinization procedures and embedded in paraffin blocks. Tissue cross sections of 5 microns from the paraffin blocks were placed on poly-l-lysine-coated slides. The cross sections were then subjected to the following procedures. They were kept in a drying oven at +80°C for 20 min, passed through a pure xylol series for 20 min and then through an ethyl alcohol series (99.5%, 96%, 90%, 80%, 70%) for 15 min suspended in distilled water, and incubated with 3% H_2_O_2_ in methyl alcohol for 10 min. This was followed by incubation for 15 min in 10% citrate buffer, pH 6.0, in a microwave oven (750 mw), and then at room temperature for 20 min. After suspension in PBS for 5 min, they were incubated with HRP blocking agent for 10 min and then in a water bath at +38°C with the tissue antibody (primary antibody eNOS mouse monoclonal IgG, Santa Cruz Biotechnology) for 30 min. Following suspension in PBS for 5 min they were incubated with biotinylated rabbit anti-mouse antibody in a water bath at +38°C for 10 min. After a further 5 min in PBS, they were incubated with Streptavidin Peroxidase in a water bath at +38°C for 10 min and then in PBS for 5 min and finally with the chromogen AEC (3-amino-9-ethyl carbazole) in a water bath at +38°C for 10 min. The sections were washed for 1-2 min in distilled water; excess water was removed, but the sections were not totally dried. One or two rounds of contrast staining were performed with Mayer hematoxylin, followed by a further washing for 1-2 min in distilled water; again, excess water was removed but the sections were not totally dried. The stained cross sections were covered with lamellae to create permanent preparations, which were examined under a light microscope and photographed. Immunohistochemical staining was assessed according to the intensity and extent of staining: 0: no staining; +1: slight; +2: moderate; +3: intense.

### 2.7. Statistics

Data are expressed as the means ± SEM. In some experiments, the statistical significance of differences was evaluated with Dunnett's test for multiple comparisons after a one-way analysis of variance, a probability level of *P* < 0.05 being regarded as significant. Statistical comparisons between concentration-response curves were made by means of a two-way analysis of variance (ANOVA) with a post hoc Bonferroni correction for multiple comparisons. A two-tailed value of *P* < 0.05 was considered significant.

## 3. Results

### 3.1. General

As indicated in Table 1, plasma glucose levels were significantly elevated above control in HFCS-fed rats. Plasma homocysteine and uric acid levels were significantly higher in HFCS-fed mice. Plasma total cholesterol, low density lipoprotein cholesterol, and triglyceride levels were all significantly increased in HCFS-fed rats (Table 1). Blood pressure in the HFCS-fed rat was significantly higher than that of the control (Table 2). CAPE supplementation to HFCS-fed rats almost normalized these abnormalities.

### 3.2. Contractile Responses Induced by Phenylephrine

**Figure 1:**
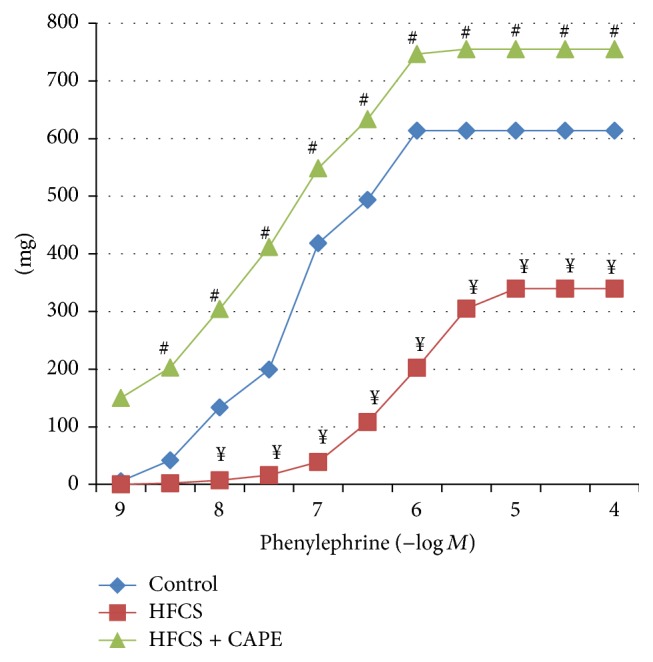
The phenylephrine dose-response curve in the rats' thoracic aorta. ^¥^ *P* < 0.05, control versus HFCS. ^#^ *P* < 0.05, HFCS versus HFCS + CAPE.

Figure 1 shows dose-response curves for the contractile responses of aortic rings to phenylephrine (10^−9^ M–10^−4^ M) in the three groups. The contractile response of aortic rings to phenylephrine in the HFCS-fed rats was much smaller than that of the control. In contrast, the phenylephrine-induced contractile response in the CAPE-treatment HFCS-fed rats was greater than that of the HFCS-fed rats. The phenylephrine-aortic contractions did not differ between control and CAPE-treatment HFCS-fed rats.

### 3.3. Relaxation Responses Induced by Acetylcholine

**Figure 2:**
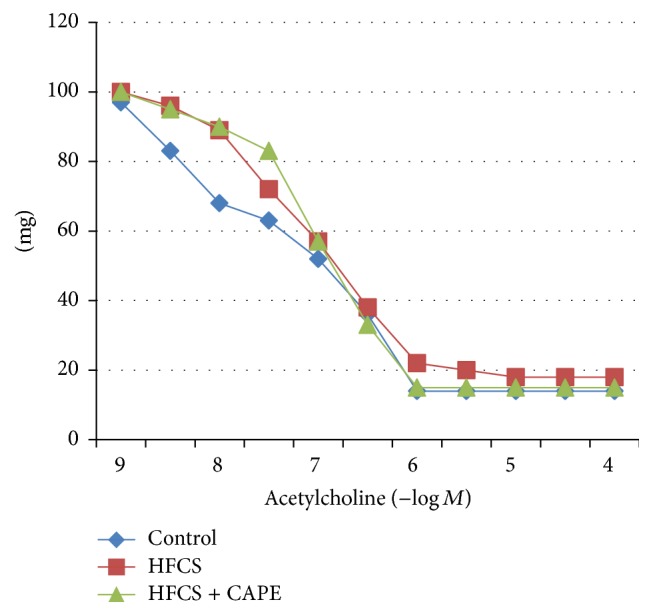
The acetylcholine dose-response curve in the rats' thoracic aorta.

The results are summarized in Figure 2. When the contraction induced by phenylephrine (10^−6^ M) had reached a plateau, acetylcholine (10^−9^ M–10^−4^ M) was added cumulatively. The relaxation caused by acetylcholine (10^−9^ M–10^−4^ M) did not differ among the three groups.

### 3.4. Immunohistochemical eNOS Values

**Figure 3:**
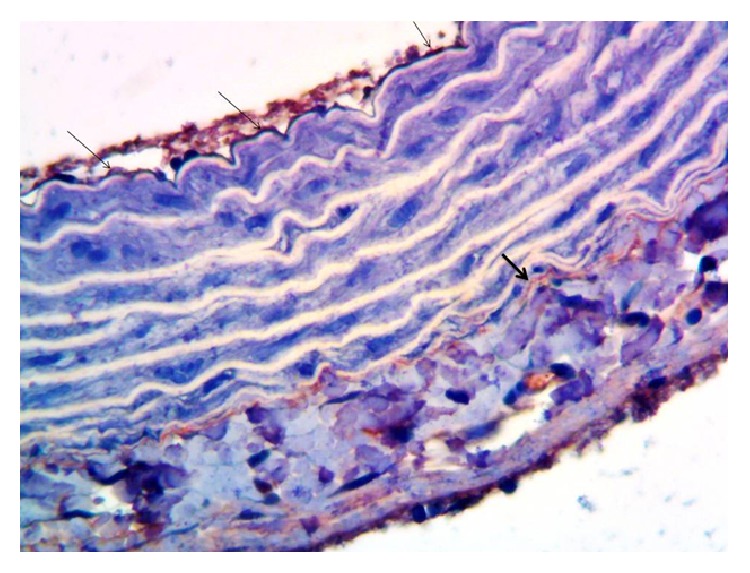
The eNOS immunoreactivity in the control group of rats' endothelium (thin arrow) and adventitia (thick arrow) of thoracic aorta, ×400.

**Figure 4:**
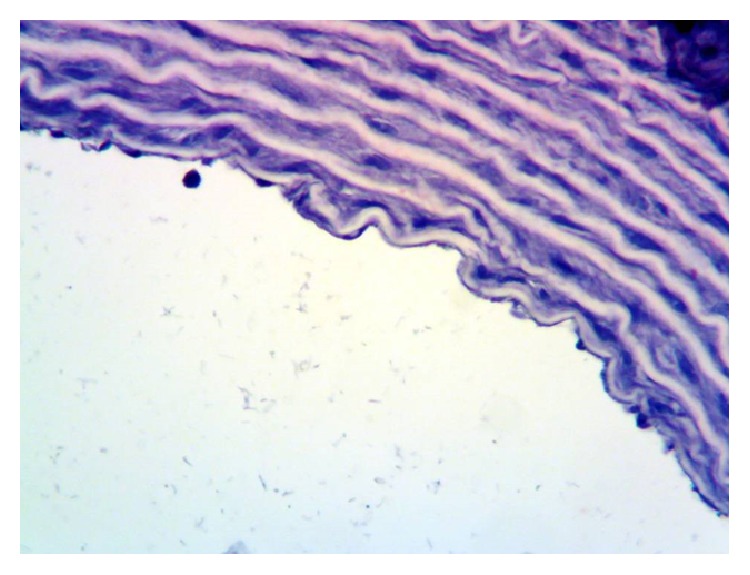
HFCS-fed group of rats' vascular endothelial tissues, eNOS immunoreactivity not observed, ×400.

**Figure 5:**
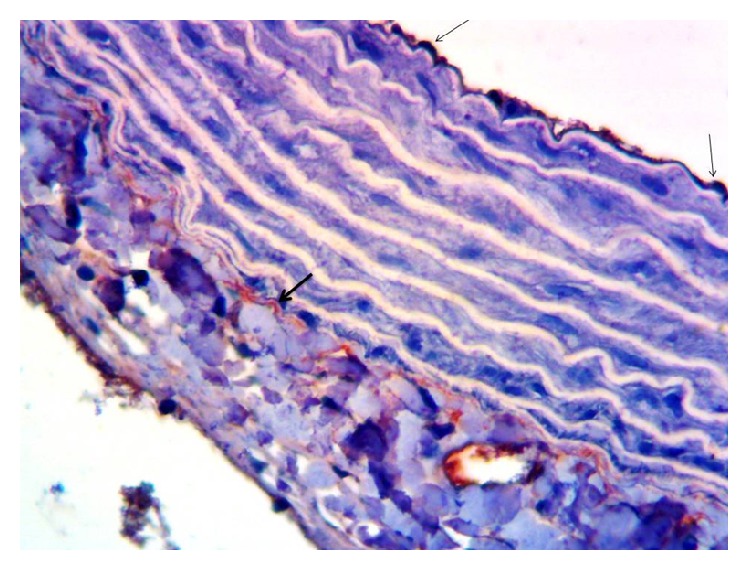
The eNOS immunoreactivity in the HFCS + CAPE treated rats' endothelium (thin arrow) and adventitia (thick arrow) of thoracic aorta, ×400.

When evaluating the rat thoracic aorta tissue as immunohistochemical, in both the endothelium and adventitia eNOS immunoreactivity was slight (+1) (Figure 3) in control rats. In vessels taken from rats fed with HFCS, eNOS immunoreaction was not observed, no staining (—) (Figure 4). In contrast, in vessels taken from rats with HFCS + CAPE group, endothelial eNOS immunoreaction was higher, moderate (+2) (Figure 5).

## 4. Discussion

In our study, compared with the control group rats, findings of hypertension, hyperglycemia, hypercholesterolemia, and hypertriglyceridemia, which are indicators of metabolic syndrome, were found in the group of rats given HFCS for 6 weeks. However, there were not significant weight gains in HFCS-fed rats. Hwang et al. demonstrated in 1987 that 66% fructose, provided in the diet, caused hypertension, hypertriglyceridemia, and hyperinsulinemia in rats [13]. It was observed that adding fructose in the diet of rodents used as laboratory animals raised blood pressure at the end of 6–8 weeks [28–30]. High fructose dietary and the way of forming metabolic syndrome vary according to the fructose concentration and the duration of feeding [31]. It has been shown that high fructose content during this time did not cause weight gain in animals. From this point, it imitates the metabolic syndrome, where no human obesity accompanies. All these findings support the results of our study. Reduction of plasma insulin levels or creation of insulin sensitization corrects the hypertension induced by fructose. For example, it was observed that after making exercises to these animals, their sensitivity to insulin had increased, insulin level decreased, and their blood pressure dropped [32]. It has been observed that many compounds that increase insulin sensitivity such as metformin or thiazolidinediones lower blood pressure [30, 33]. These findings show that insulin resistance and hyperinsulinemia cause hypertension. Many mechanisms have been suggested in the relationship of insulin resistance with hyperinsulinemia and the hypertension; it has been suggested that, among these, the activity of endothelin-1 (ET-1), angiotensin II, and thromboxane A2 (TxA2), which are the vasoconstrictor compounds of the sympathetic nervous system activation, increased [34–36]. In a survey conducted on two-month adult Wistar rats, it was found out that the body weight of the animals, which drank fructose after they were given 10% fructose within drinking water during two weeks, partially reduced but that was not found significant and the blood glucose level remained unchanged. However, peripheral vascular resistance in the fructose drinking group increased significantly [37]. In another study [38], metabolic syndrome was formed by giving 23% glucose, fructose, and sucrose within drinking water during 14 days. The animals were found with equal amounts of water to drink fructose-drinking group compared to the control animals. It was observed that the amount of drinking increased in the group given glucose and sucrose. There was no difference between the weights of the animals.

In our study, it was observed that all the above negativity was improved in the HFCS + CAPE rats. In a study supporting our results, CAPE has improved the hypertension, hypercholesterolemia, and hypertriglyceridemia findings occurring in diabetes [39]. The thesis was defended in this study that CAPE was correcting hypertension by preventing the synthesis of collagen occurring on the vessels in diabetes cases and corrected the vascular tonus. Moreover, it was defended in this study that CAPE lowered blood sugar by decreasing insulin resistance. TNF-*α* level in insulin resistance is the most important indicator. In this study CAPE significantly lowered the TNF-*α* level and returned the insulin level to normal. And also in another study, CAPE prevented this negativity in the hypertension induced by the cadmium [40].

The immunohistochemical eNOS levels obtained in our study were found significantly lower in the HFCS-fed rats compared to the control rats, and, in the HFCS + CAPE rats, eNOS levels were found significantly higher compared to the HFCS-fed rats. This may explain why high blood pressure is seen in the HFCS-fed rats. Also, having a significantly higher eNOS level in the thoracic aortas sample of the groups given CAPE as a treatment compared to HFCS-fed rats supports why the blood pressure is closer to the control rats. Probably the reasons for the decrease in the eNOS are HCFS-induced hypertension, hyperglycemia, hypercholesterolemia, hyperuricemia, and hypertriglyceridemia in our study. CAPE dropped all except uric acid. It is well known that all of them are the enemy of eNOS production. eNOS allows the endothelial NO production and NO has an important role in the control of blood pressure [41].

Another result of our study is that, in the thoracic aortic rings obtained from the rats in the HFCS-fed rats and HFCS + CAPE rats after six weeks, it was seen that acetylcholine-induced endothelium-dependent relaxation responses were identical to that of the control group, whereas in the contraction responses obtained due to phenylephrine there was a significant decrease in the HFCS-fed rats compared to the control rats and in the HFCS + CAPE, it turned to normal. As it was shown in our study, another group of researchers showed that in the aorta taken from the rats given fructose both the maximal contractile responses and the sensitivity to the agonists decreased [42]. In this study conducted by Kamata and his colleagues, five-week rats were used and three groups were formed (control, the group treated with fructose for twelve weeks and the diabetes group treated with STZ) and then their blood pressure was measured and the blood pressure of the last two groups was significantly higher than that of the control group. However, they have used the aortic strips which they have isolated in those rats and they showed that the contraction induced by noradrenaline, KCL, and serotonin significantly reduced compared to the control group. In addition, they also showed that the relaxation responses they have obtained with acetylcholine and sodium nitroprusside in these groups were the same with those of the control group. All of these findings are entirely similar to the results in our study. They have suggested that, on these findings, they have obtained the guanosine triphosphate (GTP) binding protein activity in the endothelium of the fructose-fed rats increased and thus, having bound to the adrenergic alpha-2 receptor, increased its activity and thus this led to this result. This result, in another similar study as well, suggested that the calcium release from the intracellular storage necessary for vascular contraction and the extracellular calcium influx into the cell could have reduced in diabetes; moreover, the formation of ATP as the result of any disorder in the energy production pathways of the diabetic aorta could reduce and this might cause the inhibition of the contractility [43]. When taking into consideration that the protein synthesis was also inhibited in diabetes, it would be a logical approach to think that the decrease in the responses is caused by a possible change in the contractile protein function. Indeed, in experimental diabetes, it was shown that the structure and function of actin and the microtubule could be modified as a result of glycosylation [44]. Similarly, in another study [45], the phenylephrine and KCl responses of the twelve-week-old STZ-diabetic rats' aorta strips were examined. The researchers have found that the agonist responses in diabetics significantly decreased compared to the control rats. They have suggested that the decrease in responses was caused by the diabetic neuropathy, because autonomic innervation in rat aorta was at a very low level. In addition, they have reported that the obtained findings were not related to the direct effects of hyperglycemia, either. They have shown that although the blood sugar in insulin-treated rats was still at a high level, the responses returned to normal. Considering this result, it is thought that rather than the hyperglycemia, the hyperinsulinemia was more responsible for the decrease in the responses.

When we look at the other studies in the literature, we find other studies as well which support our survey. For example, it was shown that the vascular reactivity and/or the sensitivity against various agonists decreased in chronic perfused vessel chambers [46] and STZ-diabetic [47] and alloxan- (ALL-) diabetic [48] isolated rat aorta preparates. These data suggest that the decrease in the phenylephrine contractile responses observed in our survey could be due to the decrease in the number of the alpha adrenergic receptor or in its affinity. Indeed, researches [49–52] that show the decrease in the alpha-adrenergic responses in diabetic rats prove this thesis. In other studies which also support our results, it was shown that there was no any difference in the endothelium-dependent relaxation of acetylcholine of the STZ-diabetic rat aorta compared to the control group [53–56]. In addition, in another study [57] as well, it was reported that there was no any change in the aorta of STZ-diabetic rats during the formation of the cyclic guanosine monophosphate (cGMP) which was stimulated by basal and acetylcholine. In another similar study, it was also found that there was no any change in the acetylcholine responses in the mesenteric artery of the ALL-diabetic rats [48].

In conclusion, in this study it was shown that the contraction responses to phenylephrine in the HFCS-treated rats were significantly lesser than those of the control group, whereas the relaxation responses obtained by acetylcholine were indistinguishable from those of the control rats. Also in our study it was observed that the CAPE applied for treatment purpose improved the decrease in the HFCS-related phenylephrine-induced contraction. Also, CAPE is applied to correcting the reduction in eNOS levels caused by HFCS. So it is seen that CAPE improves the disorder in the vascular contraction, occurring due to HFCS. The CAPE is the potential inhibitor of the enzymes such as ornithine carboxylase, 5-*α* reductase, protease, cyclooxygenase, lipoxygenase, xanthine oxidase, and HIV-1 integrase [19, 21–23]. It specifically and strongly inhibits the activation of the Nuclear Factor Kappa-B, which is a nuclear transcription factor [24]. In our thesis, we think that these features of CAPE improve the metabolic syndrome symptoms occurring in the rats treated with corn syrup high fructose and the disorders occurring in the vessels. However, further studies are needed which will clarify this situation.

## Figures and Tables

**Table 1:** Levels of various biochemical parameters in blood serum of rats in this study.

Groups	Glukoz mg/dL	Cholesterol mg/dL	HDL mg/dL	LDL mg/dL	Triglyceride mg/dL	Uric acid mg/dL	Homocysteine mg/dL
Control	133 ± 5.32	49.16 ± 4.91	15.06 ± 3.06	5.75 ± 0.91	29.33 ± 11.77	0.73 ± 0.60	16.63 ± 9.50
HFCS	151 ± 5.13^^*¥*^^	67.50 ± 6.25^*¥*^	18.09 ± 1.05	11.75 ± 2.66^*¥*^	64.50 ± 11.71^*¥*^	1.28 ± 0.17^*¥*^	55.21 ± 9.17^*¥*^
HFCS + CAPE	121 ± 13.94^**#**^	59.50 ± 2.66^#^	15.35 ± 1.96	9.1 ± 1.57^**#**^	58.33 ± 9.41^*¥*^	1.31 ± 0.19	24.53 ± 8.80^#^

^*¥*^ *P* < 0.05, control versus HFCS.

^#^ *P* < 0.05, HFCS versus HFCS + CAP.

**Table 2:** Blood pressure values in the rats in this study.

Groups	First day (mmHg)	Forty-second day (mmHg)
Control	108.83 ± 3.37	110.83 ± 2.22
HFCS	108.17 ± 3.76	130.17 ± 8.03^**¥**^
HFCS + CAPE	105.33 ± 3.77	108.83 ± 2.31^**#**^

^*¥*^ *P* < 0.05, control versus HFCS.

^#^ *P* < 0.05, HFCS versus HFCS + CAPE.
